# Radiotherapy for painful benign skeletal disorders: Results of a retrospective clinical quality assessment

**DOI:** 10.1007/s00066-019-01514-w

**Published:** 2019-08-27

**Authors:** Nderim Juniku, Oliver Micke, M. Heinrich Seegenschmiedt, Ralph Muecke

**Affiliations:** 1 Radiotherapy RADIO-LOG, Passau, Germany; 2 Department of Radiotherapy and Radiation Oncology, Franziskus Hospital Bielefeld, Bielefeld, Germany; 3 Radiotherapy Osnabrück, Osnabrück, Germany; 4 Radiotherapy RheinMainNahe, Bad Kreuznach, Germany; 5 Department of Radiotherapy and Radiation Oncology, Marien Hospital Herne, Ruhr, University Bochum, Bochum, Germany

**Keywords:** Low Dose Radiotherapy, Health Service Research, Arthrosis, Enthesopathy, Visual-Analogue-Scale, Niedrigdosierte Bestrahlung, Versorgungsforschung, Arthrose, Enthesiopathie, Visual-Analog-Skala

## Abstract

**Purpose:**

The aim of this retrospective clinical quality assessment was to evaluate the efficacy of low-dose radiotherapy (RT) for painful benign skeletal disorders.

**Methods:**

Patients with different painful benign skeletal disorders (arthrosis and enthesopathies) were recruited for this retrospective clinical quality assessment between January 2014 and December 2015. RT was applied with a linear accelerator. Single doses of 0.5 Gy (total dose 3.0–5.0 Gy) were used. Pain was measured before and immediately after RT (early response) by a 10-point visual analogue scale (VAS). We defined a VAS score of 0–2 as a good response. Pain relief was measured during follow-up.

**Results:**

A total of 598 evaluable patients (394 females, 204 males) with a mean age of 61.4 years (range 33–81 years) were recruited. The median VAS score was 7.0 (interquartile range [IQR] 2) before treatment and 5.0 (IQR 4) upon completion of RT (*p* < 0.001). A good response was achieved upon completion of RT in 83 patients (13.9%), with a median follow-up of 38 months (range 29–47 months) in 373 patients (62.4%; *p* < 0.001). In general, RT had a better effect on enthesopathies than on arthrosis.

**Conclusion:**

Low-dose RT is a very effective treatment for the management of painful benign skeletal disorders. Due to the delayed onset of analgesic effects, low-dose RT results in significantly improved long-term efficacy compared to the results immediately after RT. These findings confirm the results of other retrospective, prospective, and randomized trials.

## Zusammenfassung

**Hintergrund:**

Ziel dieser retrospektiven klinischen Qualitätskontrolle war es, die Wirksamkeit der niedrigdosierten Radiotherapie (RT) bei schmerzhaften benignen muskuloskeletalen Erkrankungen zu bewerten.

**Methoden:**

Zwischen Januar 2014 und Dezember 2015 wurden Patienten mit schmerzhaften muskuloskeletalen Erkrankungen (Arthrosen und Enthesiopathien) für die Studie rekrutiert. Für die RT wurde ein Linearbeschleuniger verwendet. Die RT erfolgte mit Einzeldosen von 0,5 Gy und Gesamtdosen von 3,0 bis 5,0 Gy. Der Schmerz wurde vor und direkt nach der RT (frühes Ansprechen) mithilfe einer 10 Punkte umfassenden visuellen Analogskala (VAS) bewertet. VAS-Werte von 0–2 wurden als gutes Ansprechen definiert. Das Ansprechen der RT gemäß VAS wurde zur Nachsorge erneut bewertet.

**Ergebnisse:**

Insgesamt wurden 598 auswertbare Fälle von Patienten (394 Frauen, 204 Männer) mit einem Durchschnittsalter von 61,4 Jahren (Spanne: 33–81) in die Auswertung einbezogen. Der mediane VAS-Wert betrug 7,0 (Interquartilsabstand, IQR: 2) vor der Behandlung und 5,0 (IQR: 4) unmittelbar nach Beendigung der RT (*p* < 0,001). Bei 83 Patienten (13,9%) wurde sofort nach Abschluss der RT ein gutes Ansprechen erzielt, nach einer medianen Nachbeobachtungszeit von 38 Monaten (29–47) zeigte sich ein gutes Ansprechen bei 373 Patienten (62,4%; *p* < 0,001). Die RT führte bei den Enthesiopathien zu signifikant besseren Ergebnissen im Vergleich zu den Arthrosen.

**Schlussfolgerungen:**

Die niedrig dosierte RT ist wirksam in der Behandlung von schmerzhaften benignen muskuloskeletalen Erkrankungen. Durch die zeitlich verzögert ablaufenden radiobiologischen Effekte führt die niedrig dosierte RT zu einer signifikant besseren Langzeitwirkung im Vergleich zu den Ergebnissen unmittelbar direkt nach der RT. Die Resultate sind vergleichbar mit denen aus publizierten retrospektiven, prospektiven und randomisierten Studien.

## Background

Unchanged low-dose radiotherapy (RT) for benign diseases accounts for 8–10% of all RT procedures in Germany. As many as 70% of these indications represent painful disorders in the locomotor system [1].

Previous radiobiological studies have shown that low doses of radiation can favorably influence various inflammatory pathways and immune components, such as endothelial cells, mononuclear and polynuclear leukocytes, and macrophages [2].

In clinical practice, single doses of 0.5 to 1.0 Gy and total doses of 3.0 to 6.0 Gy per series are used. The aims of this retrospective clinical quality assessment were to analyze the therapeutic effect of low-dose irradiation immediately after completion of RT and during follow-up, and to identify possible prognostic factors in patients with painful arthrosis and enthesopathies.

## Methods

### Patients and treatment

Between January 2014 and December 2015, patients with painful arthrosis and enthesopathies were recruited for this retrospective clinical quality assessment. All patients provided informed consent regarding radiotherapy and participation in this clinical quality assessment prior to enrollment. RT was applied with a linear accelerator using 6 and 15 MV photon fields. Single doses of 0.5 Gy were administered five times a week, for a total dose of 3.0–5.0 Gy.

Pain was measured before and immediately after RT (early response) using a 10-point visual analogue scale (VAS; 0, no pain to 10, strongest pain) [3]. A VAS score of 0–2 is comparable to the “von Pannewitz” score pain-free and significantly improved; therefore, we defined a VAS score of 0–2 as a good response following RT. Pain relief was measured during follow-up.

The assessment of long-term efficacy was carried out by telephone survey. All 598 patients were available by phone. All results were recorded in an Excel (Microsoft Corporation, Redmond, WA, USA) spreadsheet and then transferred to SPSS (IBM Corporation, Armonk, NY, USA) for evaluation after completion of the survey.

### Statistical analysis

All data were stored and analyzed using the SPSS statistical package 23.0 (IBM Corp., Armonk, NY, USA). Descriptive statistics were computed for continuous and categorical variables, including median and interquartile ranges (IQR) of ordinal variables, mean and standard deviations of continuous variables, and frequencies and relative frequencies of categorical factors. The Wilcoxon signed-rank test was used to test for differences in continuous and categorical variables within the groups. To test for between-group differences, the Mann–Whitney U test was used for continuous variables and Fisher’s exact test for categorical variables, as appropriate. All *P*-values were two-sided statistical tests, and *P* < 0.05 was considered significant. A Cox proportional hazards model was used for multivariate analysis to assess the independence of pain on prognostic factors for a good response at follow-up.

## Results

### Patients

A total of 598 evaluable patients (394 women, 204 men) with a mean age of 61.4 years (range 33–81 years) were recruited for the study. The following diagnoses were given: calcaneodynia (*n* = 194), shoulder syndrome (*n* = 135), arthrosis of the hand including rhizarthritis (*n* = 95), elbow syndrome (*n* = 60), trochanteric syndrome (*n* = 54), gonarthrosis (*n* = 30), and coxarthrosis (*n* = 30). A total of 507 patients were pre-treated prior to RT, including local injections, physiotherapy, NSAIDs, and shoe insoles. Ninety-one patients were irradiated without previous treatment. Patient characteristics are summarized in Table 1.

**Table 1:** Patient and treatment characteristics

Diagnosis	Number	Mean age, years (range)	Female/male	One series	Two series	Three series	Median follow-up, months (range)
Calcaneodynia	194	57.7 (38–79)	137/57	112	75	7	38 (29–46)
Shoulder syndrome	135	65.7 (46–81)	73/62	72	54	9	39 (30–46)
Arthrosis of the hand	95	65.4 (35–80)	69/26	51	40	4	39 (30–45)
Elbow syndrome	60	52.9 (33–75)	33/27	31	26	3	38 (29–44)
Trochanteric syndrome	54	63.7 (41–78)	45/9	33	16	5	36.5 (30–45)
Gonarthrosis	30	62.4 (46–80)	17/13	19	10	1	37.5 (30–44)
Coxarthrosis	30	64.9 (51–78)	20/10	21	8	1	38 (30–47)
All patients	598	61.4 (33–81)	394/204	339	229	30	38 (29–47)

### Treatment and series

RT was fractionated into 6 × 0.5 Gy in 34 patients and with 10 × 0.5 Gy in 564 patients. The standard was fractionation of 10 × 0.5 Gy. In 34 cases, due to individual reasons, RT was fractionated as 6 × 0.5 Gy. RT was performed as one series in 339 patients, two series in 229 patients, and three series in 40 patients based on insufficient remission of pain. RT of shoulder, hip, knee joints, and trochanteric syndrome was performed with opposing photon fields, and RT of the heels, hands, and elbows was performed with single photon fields.

### VAS before and immediately upon completion of RT

The median VAS score was 7.0 (IQR 2) before treatment and 5.0 (IQR 4) immediately upon completion of RT (*p* < 0.001). The results of RT for patients with different diagnoses are summarized in Table 2.

**Table 2:** Median VAS scores before and at completion of RT (Interquartile Range)

Diagnosis	Before RT (interquartile range)	At completion of RT (interquartile range)	*P*-value
Calcaneodynia	7.0 (2)	5.0 (3)	<0.001
Shoulder syndrome	7.0 (2)	5.0 (3)	<0.001
Arthrosis of the hand	7.0 (3)	5.0 (4)	<0.001
Elbow syndrome	8.0 (2)	6.0 (4)	<0.001
Trochanteric syndrome	8.0 (2)	4.0 (4)	<0.001
Gonarthrosis	7.0 (1)	6.0 (4)	<0.001
Coxarthrosis	8.0 (2)	6.0 (4)	0.002
All patients	7.0 (2)	5.0 (4)	<0.001

### VAS follow-up

Twelve weeks after radiotherapy (*n* = 339), the median VAS score was 4.0 (IQR 4). After a median follow-up of 38 months (range 29–47 months), the median VAS score was 1.0 (IQR 4; *p* = 0.001).

### Good response immediately upon completion of RT

A good response was achieved immediately upon completion of RT in 83/598 patients (13.9%) and 12 weeks after radiotherapy in 119/339 patients (35.1%; *p* < 0.001).

### Good response at follow-up

A good response was achieved within a median follow-up of 38 months in 373/598 patients (62.4%). This is a significant improvement over the results immediately upon completion of RT and 12 weeks after RT (*p* < 0.001). Results for the different diagnoses are given in Table 3.

**Table 3:** Proportion of good responses at completion of RT and follow-up

Diagnosis	At completion of RT, %	At follow-up, %	*P*-value
Calcaneodynia	13.9 (27/194 patients)	85.6 (166/194 patients)	<0.001
Shoulder syndrome	17.7 (24/135 patients)	53.3 (72/135 patients)	<0.001
Arthrosis of the hand	9.5 (9/95 patients)	50.5 (48/95 patients)	<0.001
Elbow syndrome	15 (9/60 patients)	70 (42/60 patients)	<0.001
Trochanteric syndrome	16.7 (9/54 patients)	57.4 (31/54 patients)	<0.001
Gonarthrosis	10 (3/30 patients)	20 (6/30 patients)	0.317
Coxarthrosis	6.7 (2/30 patients)	26.7 (8/30 patients)	0.014
All patients	13.9 (83/598 patients)	62.4 (373/598 patients)	<0.001

### Rate of recurrence after a good response during follow-up

Compared to immediately after RT, 15 of the 83 patients (18.1%) with a good response 12 weeks after the onset of RT exhibited pain recurrence. Compared to 12 weeks after the onset of RT, 25 of the 119 patients (21%) with a good response within the 38-month follow-up exhibited pain recurrence.

### Comparison of results for enthesopathies and arthrosis

Treatment results comparing enthesopathies and arthrosis are given in Table 4. In general, RT had a better effect on enthesopathies than on arthrosis.

**Table 4:** VAS scores and good responses in enthesopathies versus arthrosis at RT completion and follow-up

	Enthesopathies (*n* = 443)	Arthrosis (*n* = 155)	*P*-value
Median VAS at RT completion (interquartile range)	5.0 (3)	5.0 (4)	0.985
Median VAS at follow-up (interquartile range)	0.0 (3)	4.0 (7)	<0.001
Good response at RT completion, % (*n*/*N*)	15.6 (69/443)	14 (9/155)	0.054
Good response at follow-up, % (*n*/*N*)	70.2 (331/443)	40 (62/155)	<0.001

### Comparison of sex

Results regarding biological sex are given in Table 5. Efficacy immediately after RT (early response) was better in men than in women, but no significant difference was found at follow-up.

**Table 5:** Influence of sex on VAS scores and good responses at RT completion and follow-up

	Female (*n* = 394)	Male (*n* = 204)	*P*-value
Median VAS at RT completion (interquartile range)	5.0 (4)	5.0 (4)	0.035
Median VAS at follow-up (interquartile range)	1.0 (5)	0.5 (5)	<0.001
Good response at RT completion, % (*n*/*N*)	11.9% (47/394)	17.6% (36/204)	0.037
Good response at follow-up, % (*n*/*N*)	60.2% (237/394)	66.7% (136/204)	0.780

### Multivariate analysis

The multivariate Cox regression analysis including sex, age, pre-treatment, disease group (enthesopathies or arthrosis), and number of treatment series revealed male gender (*p* = 0.031) and patients with enthesopathies (*p* < 0.001) as significant prognostic factors for a good response at follow-up (Table 6).

**Table 6:** Multivariate analysis of prognostic factors for a good response at follow-up

Factor	Distribution	Number	Good response at follow-up (%)	*P*-value
Sex	Female/male	394/204	60.2/66.7	0.031
Age	<62 years/≥62 years	309/289	65/59.5	0.374
Pre-treatment	Yes/No	507/91	62.7/60.4	0.920
Disease group	Entesopathy/arthrosis	443/155	70.2/40	<0.001
Number of series	One/two/three	339/229/30	64.3/59.4/63.3	0.774

### Comparison of fractionation

With regard to fractionation, we found no significant differences between 6 × 0.5 Gy and 10 × 0.5 Gy.

No side effects were observed.

## Discussion

The results of our retrospective clinical quality assessment confirmed the results of recently published retrospective and prospective randomized studies showing a good analgesic effect of low-dose RT in patients with enthesopathies or arthrosis [4–13]. The pathophysiological mechanisms of pain relief after RT are most likely multifactorial. Radiobiological experiments clearly indicate that low-dose RT has an anti-inflammatory effect due to the modulation of a variety of inflammatory pathways and the influence on cellular components, such as endothelial cells, mononuclear and polynuclear leukocytes, and macrophages. An influence of the vascular endothelium, with improved tissue perfusion, destruction of inflammatory cells (especially lymphocytes) and release of cytokines and proteolytic enzymes, modulation of the autonomic nervous system, a change in tissue pH, and increased membrane permeability, has been demonstrated previously in experimental studies [2].

Compared to the results immediately after RT, we observed a significant improvement in efficacy in the long term, confirming the results of other comparable studies. The relevant radiobiological mechanisms could cause the delayed clinical onset [2, 12, 13].

Similar to recently published results, our data show a significantly better response to RT in patients with enthesopathies than in patients with arthritis [12, 13]. Arthroses reflect irreversible pathological processes in which cartilaginous and bony destruction occurs that cannot be reversed by radiotherapy. This irreversible destruction can trigger inflammatory processes, which then appear clinically as activated osteoarthritis with pain and swelling. During these episodes of pain, low-dose RT may be helpful due to known radiobiological mechanisms. The arthrosis itself remains, despite the improvement in pain. Therefore, the analgesic effect on arthrosis is only moderate compared to the analgesic effect on enthesopathies.

Furthermore, the significantly better response to RT in men compared to women confirms the well-known evidence of general differences between men and women in terms of pain perception and pain assessment [14].

With regard to the number of treatment series in the same treatment region, the results of the patients who received two or three series are worse. Therefore, in the future, we will make the indication for low-dose radiotherapy strict in the case of repeated treatment series, because non-responders do not substantially benefit from additional treatment series.

A possible placebo effect of low-dose RT for the treatment of pain cannot be completely ruled out. Two randomized, blinded, and sham-controlled trials on knee and hand joint osteoarthritis were published recently and showed no significant difference in remission for RT groups compared to placebo groups [15, 16]. Despite the good study design, both studies are open to critique because of low patient numbers, short follow-up (3 months), and presumption of a very optimistic prognosis for the assessment of success. In addition, approximately 50% of the included patients had experienced pain ≥5 years before RT. In the future, randomized trials should include more patients, include patients with less advanced arthrosis and shorter duration of pain, and have a longer follow-up [17].

Side effects did not occur in any of our patients. This confirms the relevant results in the literature [4–13].

With respect to the comparison of 6 × 0.5 Gy (*n* = 34) and 10 × 0.5 Gy (*n* = 564), we found no significant advantages of any of the fractionation schemes. Therefore, in the future, for radiation protection, irradiation should be performed according to the recommended German guidelines: enthesopathies and trochanteric syndrome with single doses of 0.5 to 1.0 Gy up to a total dose of 3.0 Gy, and arthrosis with single doses of 0.5 to 1.0 Gy up to a total dose of 3.0 to 6.0 Gy [4].

Due to the known cancer risk, the indication for irradiation in patients younger than 40 years should be very critical. However, in older patients, the cancer risk can be neglected because it is low compared to the improvement in quality of life.

The limitations of our evaluation are the lack of randomization and blinding. Nevertheless, the crucial strength of the analysis is the long follow-up. Importantly, our analysis shows that low-dose RT of benign musculoskeletal diseases plays a very important, tendentially increasing, role in the context of health care in Germany in view of the aging population.

## Conclusion

Low-dose RT is a very effective treatment for the management of painful benign skeletal disorders. In the present study, 62.4% of patients achieved a longer-lasting significant improvement in their quality of life. Due to the delayed onset of analgesic effects, low-dose RT results in significantly improved long-term efficacy compared to the results immediately after RT. In general, RT had a better effect on enthesopathies than on arthrosis. The results of our retrospective clinical quality assessment confirm the results of other retrospective, prospective, and randomized trials.
